# Association of prenatal and childhood environment smoking exposure with puberty timing: a systematic review and meta-analysis

**DOI:** 10.1186/s12199-018-0722-3

**Published:** 2018-07-18

**Authors:** Yiwen Chen, Qin Liu, Wenyan Li, Xu Deng, Bo Yang, Xin Huang

**Affiliations:** School of Public Health and Management, Research Center for Medicine and Social Development, Collaborative Innovation Center of Social Risks Governance in Health, Chongqing Medical University, Chongqing, China

**Keywords:** Prenatal smoking, Childhood environment tobacco smoke, Puberty timing, Systematic reviews, Meta-analysis

## Abstract

**Objectives:**

Mothers who smoke during pregnancy or while their children are small were common in some populations. Epidemiological studies have tried to detect the effect of prenatal tobacco smoke (PTS), and childhood environmental tobacco smoke (ETS) on puberty timing have not shown a consensus results. We aimed to examine current evidence and estimate the associations between PTS or/and ETS and puberty timing.

**Methods:**

Seven databases were searched from inception to May 2017. All the cohort studies examining the associations between PTS and/or ETS and puberty timing were identified. Two reviewers independently screened all studies, evaluated the quality of eligible studies, and extracted the data. The quality assessment of the eligible cohort studies was based on the Newcastle-Ottawa Scale. Risk ratio (RR), standard mean difference (SMD), and 95% confidence intervals (CIs) were calculated and pooled by CMA (Version 2.0, Biostat, Inc., USA).

**Results:**

Compared with controls, girls with PTS and ETS exposure have an earlier age at menarche (SMD − 0.087, 95% CI 0.174 to − 0.000), and similar results were found in both PTS subgroup (SMD − 0.097, 95% CI − 0.192 to − 0.002) and prospective cohort subgroup (SMD − 0.171, 95% CI − 0.253 to − 0.090). And number of boys with early voice break in PTS group was significantly increasing than non-exposed boys (RR 1.34, 95% CI 1.29 to 1.40).

**Conclusions:**

PTS exposure possibly decrease age of menarche of girls, and studies on boys were urgent needed. Appropriate and comprehensive outcome measures using unified criteria to classify puberty should be reported in future studies.

**Electronic supplementary material:**

The online version of this article (10.1186/s12199-018-0722-3) contains supplementary material, which is available to authorized users.

## Background

Advanced puberty timing has been subject to increasing interest and concern worldwide during recent years. There were evidences that early puberty was on the rise among girls in many parts of the world, such as Gambia [1], America [2–4], Western Europe [5], and for boys were inconsistent [3, 6, 7]. Early menarche in girls was a risk factor for the occurrence of morbid obesity, hypertension as well as breast and endometrial cancer [8–11]. Recent study provided strong evidence that the younger girls were at menarche, the greater was their risk of premature and early menopause [12]. Although there were less research in boys, a review suggests that early puberty was also a strong risk factor for detrimental psychosocial outcomes [13]. Factors affecting early puberty can be categorized in two distinct ways: genetically determinant [14, 15] and non-genetically determinant [16, 17].

Smoking, in some populations, has been a widely spread non-genetic exposure, both during pregnancy and childhood. And cigarette smoking exposure included three forms: prenatal tobacco smoke (PTS), childhood environment tobacco smoke (ETS), and both PTS and ETS. In Australia, up to 80% of indigenous women smoke during pregnancy in some communities [18]. Worldwide, almost half of children were exposed to ETS [19]. Results on associations between three forms of smoking exposure and puberty timing from the past decade and more years were inconsistent. The study by Fukuda 2013 [20] reported earlier menarche in relatively young daughters with PTS exposure. However, finding by Zhang 2014 [21] showed that PTS had no effect on age of menarche of daughters. Kolasa 1998 [22] reported an earlier age at menarche related to ETS exposure, while Shrestha 2011 [23] found no association between age at menarche and ETS.

Two existing reviews studied the relationship between PTS exposure and puberty. Håkonsen 2014 [24] studied on relationship between PTS and reproductive health of adolescent including pubertal development. This review qualitatively summarized the results without meta-analysis, and it concluded that results for girls were conflicting and the number of studies for boys was sparse. Yermachenko 2015 [25] conducted a meta-analysis based on both cross-sectional studies and cohort studies to study the association between PTS exposure and age of menarche. It suggested that pregnancy smoking may decrease age at menarche.

In summary, evidences about relationship between PTS or ETS with puberty timing were inconsistent and have not been reviewed systematically so far; therefore, it is necessary to conduct a systematic review and meta-analysis to identify the associations between PTS and/or ETS and puberty timing in both girls and boys. This systematic review seeks to address this association and highlight where more research might be needed.

## Methods

### Selection criteria

Inclusion criteria: we included all the cohort studies examining relationship between PTS and/or ETS with puberty timing. In this study, since PTS exposure related to pregnant women, we determined “participants” as children, adolescents, and pregnant women; “exposures” as PTS and/or ETS; “control” as not exposing to PTS and/or ETS; “outcome measures” as the number of early puberty events and the age at puberty events.

Exclusion criteria: (1) not relevant to early puberty timing or precocious puberty; (2) other language except English and Chinese; (3) repetitive research (different articles published from the same study were considered as one study).

### Search strategy

We searched publications from inception to May 2017 by an electronic search among seven databases including PubMed, ISI Web of science, OVID, EBSCO, VIP Database for Chinese Technical Periodicals, WangFang Data and Chinese National Knowledge Infrastructure databases, using both the MeSH terms and free terms “puberty” or “puberty timing” or “pubertal timing” or “pubertal development” or “precocious puberty” or “sexual precocity” or “sexual prematurity” or “sexual maturation” or “menarche” or “Tanner stages” or “thelarche” or “pubarche” or “spermarche” or “spermatorrhea” or “nocturnal emission” or “testis”, in combination with “maternal exposure” or “prenatal exposure” or “prenatal smoking” or “prenatal tobacco smoke” or “PTS” or “in utero exposure” or “passive smoking” or “environmental tobacco smoke” or “ETS.” All the retrieved publications were imported into reference-managing software (EndNote, version X7, Thomson Scientific, Stamford, CT, USA) to complete the duplicate check.

### Data screening and extraction

Two reviewers (YC, WL) independently screened all the retrieved literatures by title, abstracts, and then full texts using above inclusion criteria. Cross-checking was implemented for accuracy, and differences were resolved by discussing with the third reviewer (QL) to reach an agreement.

Data extracted from included studies by using a pre-designed extraction form were as follows: (1) general information, including authors, publication year, research area; (2) study design; (3) participants characteristics, and sample size; (4) outcomes, mode, and level of tobacco exposure; (5) other factors affecting outcomes.

### Risk of bias assessment

Two reviewers (YC, WL) independently evaluated the methodology quality of each eligible study according to a pre-established assessment form based on Newcastle-Ottawa Scale [26], which distributed a score of total 9 points for each study following the criteria: 4 items for selection, 1 item for comparability, and 3 items for outcome assessment. In selection and outcome categories, at most one score can be awarded to a study for each item, but for comparability, two scores can be awarded. Studies were divided into three grades by total scores, including grade A (scored 7–9, high quality), grade B (scored 4–6, moderate quality), and grade C (scored 0–3, low quality) [27]. Differences were resolved by consulting with a third reviewer (QL).

### Statistical analysis

Meta-analysis was conducted by Comprehensive Meta-Analysis (Version 2.0, Biostat, Inc., USA). The outcomes of continuous and dichotomous variables were estimated as standardized mean difference (SMD) and relative risk (RR) with 95% confidence intervals (CIs), respectively. Heterogeneity among the results of the included studies was checked by chi-square based *Q* test and *I*^2^ test. When *p* < 0.05 or *I*^2^ > 50%, heterogeneity was considered and random effects model was used. Otherwise, Manter–Haenszal fixed-effects model was used. Results went for statistically significant when *p* value less than 0.05. We qualitatively described the main findings of included studies whose data cannot be extracted or which cannot be included in meta-analysis.

We conducted subgroup analysis based on exposure time (PTS and ETS), different PTS exposure levels in girls (1–9cigarettes/day, 10–19 cigarettes/day, and ≥ 20 cigarettes/day), cohort category (prospective cohort study and retrospective cohort study), and different definitions of early menarche. For stabilization of the results, we used the leave-one-out approach to conduct sensitivity analysis of all the outcome analyses. Since the amount of included studies did not reach the quantity requirement, we did not estimate the publication bias [28].

## Results

### Search results

**Fig. 1:**
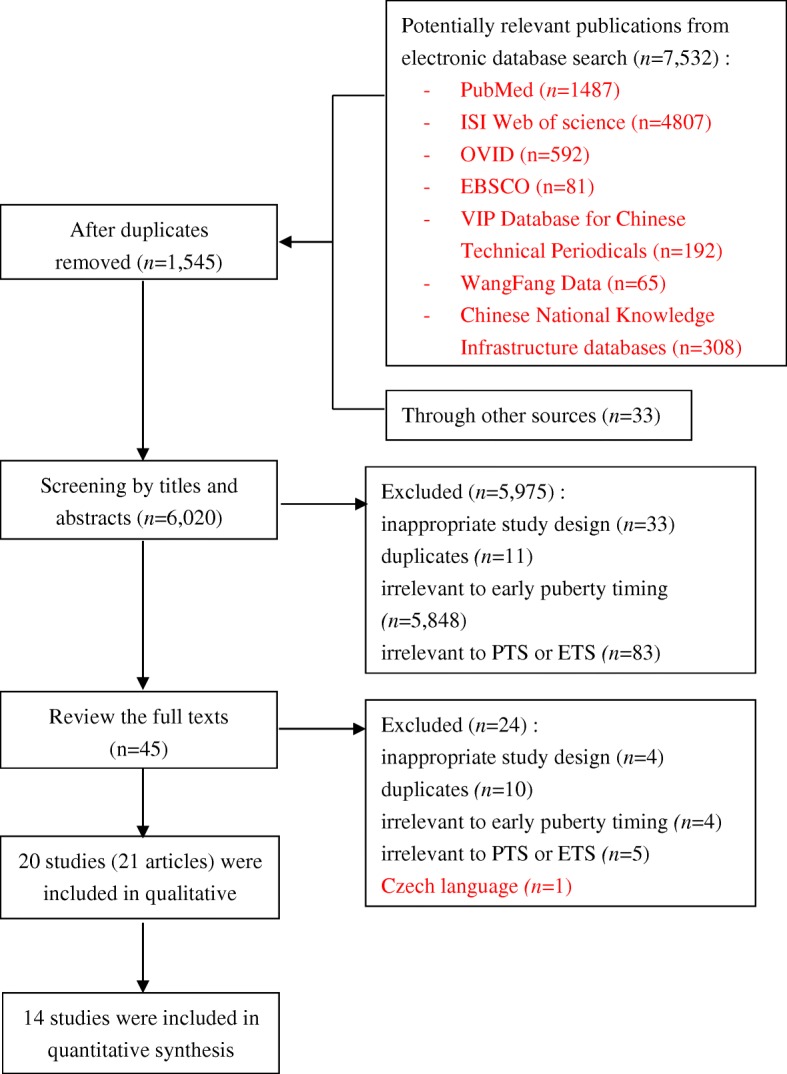
Flow diagram of the literature search

Among 7532 records identified from the seven databases and 30 records tracked from the correlative references, a total of 20 studies reported in 21 articles [23, 29–48] met the inclusion criteria were included in the qualitative synthesis. Of which, six studies cannot be included in meta-analysis; therefore, 14 studies were included in the meta-analysis finally through a strict screening process (Fig. 1).

### Characteristics of included studies

Table 1 presents the main characteristics of included studies. All the included studies were published between 2004 and 2017, with the sample size ranging from 698 to 98,995. Among those 20 studies, 8 studies were conducted in the USA, 4 were in Denmark, 3 in UK, 2 in Australia, and the other 3 studies were conducted in Iran, Canada, and France, respectively. All studies were cohort studies, of which, 16 were prospective and 4 were retrospective cohorts. Thirteen studies assessed age at menarche as main outcome, 5 studies reported numbers of girls with early menarche as main outcome, 1 study reported number of girls reached menarche, 4 studies reported breast development, 4 studies reported pubic hair development, and another 3 studies reported puberty events in boys. Seventeen studies focused only on girls, 2 studies were only on boys, and the other 1 included both boys and girls. Data collection methods in included studies contained questionnaires, clinical records, face-to-face interviews, and body measurements. The exposure type in included studies contained PTS exposure only, ETS exposure only, and both PTS and ETS exposure. For the PTS exposure, the levels of exposure were also extracted when possible.

**Table 1:** Characteristics of Included Studies

Study	Country	Gender	Age at baseline/age at follow-up surveys	End of follow-up	Sample size^a^ (expose/control^b^)	Exposure type^c^	PTS exposure level	Primary outcomes	Cohort category
Håkonsen 2013 [29]	Denmark	Boys	Prenatal/18–21 years old	18–21 years old	858/1387	PTS	Past smoker^d^ 1–9 cigs/day 10–14 cigs/day ≥ 15 cigs/day	Age at acne Age at voice break Age at regular shaving Age at first nocturnal emission	Prospective
Ernst 2012 [30]	Denmark	Girls	Prenatal/19–21 years old	Onset of menarche	129/220	PTS	1–9 cigs/day ≥ 10 cigs/day	Age at menarche	Prospective
Windham 2008 [31]	USA	Girls	Prenatal/ (1) up to 7 or 8 years old (2) 20s or 27–33 years	Onset of menarche	832/722	PTS and ETS	< 10 cigs/day 10–19 cigs /day ≥ 20 cigs/day	Age at menarche	Prospective
Ferris 2010 [32] Terry 2009 [33]	USA	Girls	Prenatal/ (1) up to 7 years old (2) 38–46 years old	Onset of menarche	C1: 98/150 C2: 90/61 C3: 95/61	PTS and ETS	1–9 cigs/day 10–19 cigs/day ≥ 20 cigs/day	No. of girls with early menarche^e^	Prospective
Behie 2015 [34]	Australia	Girls	Prenatal/ 12–13 years old	12–13 years old	222/1271	PTS	Smoking most days; smoking occasionally	Age at menarche No. of girls reached menarche	Prospective
Windham 2004 [35]	USA	Girls	Prenatal/ (1) 5 years old (2) 9–11 years old (3) 15–17 years old	16 or 17 years old	C1: 508/214, C2: 162/214, C3: 417/214	PTS and ETS	1–9 cigs/day 10–19 cigs/day ≥ 20 cigs/day	No. of girls with early menarche^f^	Prospective
Tehrani 2014 [36]	Iran	Girls	Prenatal/ evaluated once every 3 years	Onset of menarche	34/367	ETS	Not mentioned	Age at menarche	Prospective
Rubin 2009 [37]	UK	Girls	Prenatal/evaluated once yearly from 8 to 11 years	11 years old	368/2159	PTS	Not mentioned	No. of girls with early menarche^g^	Prospective
Shrestha 2011 [23]	Denmark	Girls	Prenatal/(1) 15–18 years old (2) 18–21 years old	Onset of menarche	656/970	PTS and ETS	Stop smoking sometimes during pregnancy; 1–9 cigs/day; ≥ 10 cigs/day	Age at menarche	Prospective
Maisonet 2010 [38]	UK	Girls	Prenatal/8–14 years old	Onset of menarche	647/2657	PTS	Not mentioned	Age at menarche Age at breast development stage ≥ 2 and ≥ 3 Age at pubic hair development stage≥2 and ≥ 3	Prospective
Fried 2001 [39]	Canada	Boys and girls	Prenatal/evaluated once yearly up to 8 years, thereafter, every 3 years	13–16 years old	Not mentioned	PTS	Up to 16 mg nicotine/day; ≥ 16 mg nicotine/day	Age at menarche Age at breast began to develop Age at start shaving Age at voice break	Prospective
Wang 2012 [40]	USA	Girls	Prenatal/evaluated once yearly from 10 to 15 years old	Onset of menarche	21/284	PTS	Not mentioned	Age at menarche Age of breast developmentstage1 or 2 and 3 Age of pubic hair developmentstage1 or 2 and 3	Prospective
Flom 2017 [41]	USA	Girls	Prenatal/ infancy, early childhood and adulthood	Onset of menarche	647/443	PTS	Not mentioned	No. of girls with early menarche^f^	Prospective
Dossus 2012 [42]	France	Girls	40–65 years old/ Prenatal	–	802/75,171	PTS and ETS	Not mentioned	Age at menarche	Retrospective
Hart 2009 [43]	Australia	Girls	Prenatal/2, 6, 8, 10, 13/14, and 16/17 years old	Onset of menarche	47/184	PTS	Not mentioned	Age at menarche No. of girls with breast development stage3 No. of girls with pubic hairdevelopmentstage2/3	Prospective
Morris 2010 [44]	UK	Girls	16–30 years old/prenatal	–	630/5710	PTS	Not mentioned	Age at menarche	Retrospective
D’Aloisio 2013 [45]	USA	Girls	Not mentioned/prenatal	–	11,029/21,067	PTS	Not mentioned	Age at menarche	Retrospective
Ravnborg 2011 [46]	Denmark	Boys	Not mentioned/prenatal	–	PTS:1385/2101 ETS:769/1332	PTS and ETS	Not mentioned	No. of boys with early voice break No. of boys with early growth of penis No. of boys with early pubic hair development	Retrospective
Carter 2014 [47]	USA	Girls	Prenatal/14 years old	14 years old	168/97	PTS	Not mentioned	Age at menarche	Prospective
Liu 2010 [48]	USA	Girls	Prenatal/1,4,7,18 years old	Onset of menarche	486/308	PTS	1–19 cigs/day ≥ 20 cigs/day	No. of girls with early menarche^h^	Prospective

^a^*PTS* prenatal tobacco smoke, *ETS* childhood environment tobacco smoke. *C1* PTS/control, *C2* ETS/control, *C3* both PTS and ETS/control

^b^Control group: non-smoker group

^c^Exposure type included 3 types:(1) PTS, (2) ETS, (3) both PTS and ETS

^d^Past smoker, included women who smoked before pregnancy or stopped sometime before 36th gestational week

^e^Early puberty occurred was defined according to age at menarche, ≤ 12 years for early menarche

^f^Early puberty occurred was defined according to age at menarche, < 12 years for early menarche

^g^Early puberty occurred was defined according to age at menarche, ≤ 11 years for early menarche

^h^Early puberty occurred was defined according to age at menarche, < 11 years for early menarche

### Risk of bias in included studies

Thirteen out of 20 included studies were evaluated as high-quality research, scored 7–9, while another 7 were evaluated as moderate quality research, scored 6 (Table 2). All studies had adequate representativeness of the exposed cohort, exposure ascertainment, and selection of control. All outcomes of interest were not presented at the start of prospective cohort study. For comparability, 8 out of 20 studies considered both the main confounding factors and others, like maternal BMI and maternal age at menarche. Outcome assessments of all studies were satisfied. Subjects of 12 prospective studies were followed until the outcomes occurred, while the other 4 studies were not followed up sufficiently. Loss is inevitable in cohort study and 7 out of 16 prospective cohort studies had a rate of lost to follow-up below 20%.

**Table 2:** The Newcastle-Ottawa scale (NOS) for assessing the methodology quality of cohort studies

Study	Selection				Comparability	Outcome				Score^a^
	Representativeness of the exposed cohort	Selection of control	Ascertainment of exposure	Demonstration that outcome of interest was not present at start of study	Comparability of cohorts on the basis of the design or analysis	Assessment of outcome	Was followed up long enough for outcomes to occur	Adequacy of follow-up of cohorts	Number of loss/*n*	
Håkonsen 2012 [29]	1	1	1	1	2	1	1	0	2906/5716	8
Ernst 2012 [30]	1	1	1	1	2	1	1	0	Not mentioned	8
Windham 2008 [31]	1	1	1	1	0	1	1	0	Not mentioned	6
Ferris 2010 [32]	1	1	1	1	2	1	1	0	466/841	8
Behie 2015 [34]	1	1	1	1	2	1	0	0	953/2446	7
Windham 2004 [35]	1	1	1	1	2	1	1	1	9/1003	9
Tehrani 2014 [36]	1	1	1	1	0	1	1	0	Not mentioned	6
Rubin 2009 [37]	1	1	1	1	2	1	0	1	413/2940	8
Shrestha 2011 [23]	1	1	1	1	2	1	1	0	2248/5427	8
Maisonet 2010 [38]	1	1	1	1	0	1	1	1	1104/5842	7
Fried 2001 [39]	1	1	1	1	0	1	0	1	38/190	6
Wang 2012 [40]	1	1	1	1	0	1	1	0	281/856	6
Flom 2017 [41]	1	1	1	1	0	1	1	1	180/1314	7
Dossus 2012 [42]	1	1	1	0	2	1	1	1	NA^b^	8
Hart 2009 [43]	1	1	1	1	0	1	1	0	1068/2868	6
Morris 2010 [44]	1	1	1	0	1	1	1	1	NA	7
D’Aloisio 2013 [45]	1	1	1	0	1	1	1	1	NA	7
Ravnborg 2011 [46]	1	1	1	0	0	1	1	1	NA	6
Carter 2014 [47]	1	1	1	1	0	1	0	1	0/265	6
Liu 2010 [48]	1	1	1	1	0	1	1	1	16/810	7

^a^Low-quality research, scored 0–3; moderate quality research, scored 4–6; high-quality research, scored 7–9

^b^Loss of retrospective cohort is not available

### Data synthesis

#### Age at menarche

##### Age at menarche in PTS or ETS group

**Fig. 2:**
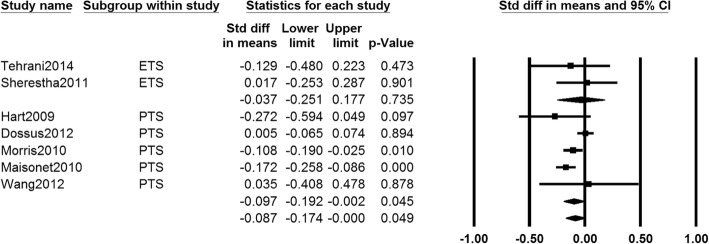
Forest plot for the age at menarche between the PTS or ETS group and control group

Data on age at menarche of girls who exposed to PTS or ETS were provided in seven studies [23, 36, 38, 40, 42–44]. The pooled data showed that age at menarche in girls with PTS and ETS exposure (SMD − 0.087, 95% CI − 0.174 to − 0.000, *I*^2^ = 52.813%, 87,309 girls) was significantly lower than control group, and similar results were found in PTS subgroup (SMD − 0.097, 95% CI − 0.192 to − 0.002, *I*^2^ = 67.069%, 86,153 girls). There was no significantly difference in age of menarche between ETS subgroup (SMD − 0.037, 95% CI − 0.251 to 0.177, *I*^2^ = 0.000%, 1156 girls) and control group. The random effects model was adopted because of heterogeneity among studies (Fig. 2).

For the five studies reporting PTS exposure, we conducted the cohort-categories subgroup analysis. We found that age at menarche in prospective cohort subgroup (SMD − 0.171, 95% CI − 0.253 to − 0.090, *I*^2^ = 0.000%, 3840 girls) was significantly lower than control group. No significantly difference was found in age of menarche between retrospective cohort subgroup (SMD − 0.128, 95% CI − 0.194 to 0.062, *I*^2^ = 76.303%, 82,313 girls) and control group (see Additional file 1: Figure S1). In the sensitivity analysis, the pooled results altered when removing Maisonet 2010 [38] (SMD − 0.046, 95% CI − 0.097 to 0.004, 84,005 girls).

Two included studies cannot be included in the meta-analysis. D’Aloisio 2013 [45] divided the children into five age groups according to their age at menarche (≤ 10, 11, 12–13, 14, ≥ 15 years old) and compared the relative risk ratios for early and late menarche in association with prenatal smoking exposure. It did not report the exact age at menarche for the girls and cannot be included in the meta-analysis. It found that PTS associated with early menarche (RR 1.13, 95% CI 1.03 to 1.24, 32,095 girls).

Carter 2014 [47] found no significant relationship between age at menarche and PTS (*r* 0.17, *p* > 0.05, 265 girls), but did not report the sample of girls who exposed to PTS and had onset of menarche.

##### Age at menarche in different PTS exposure levels

**Fig. 3:**
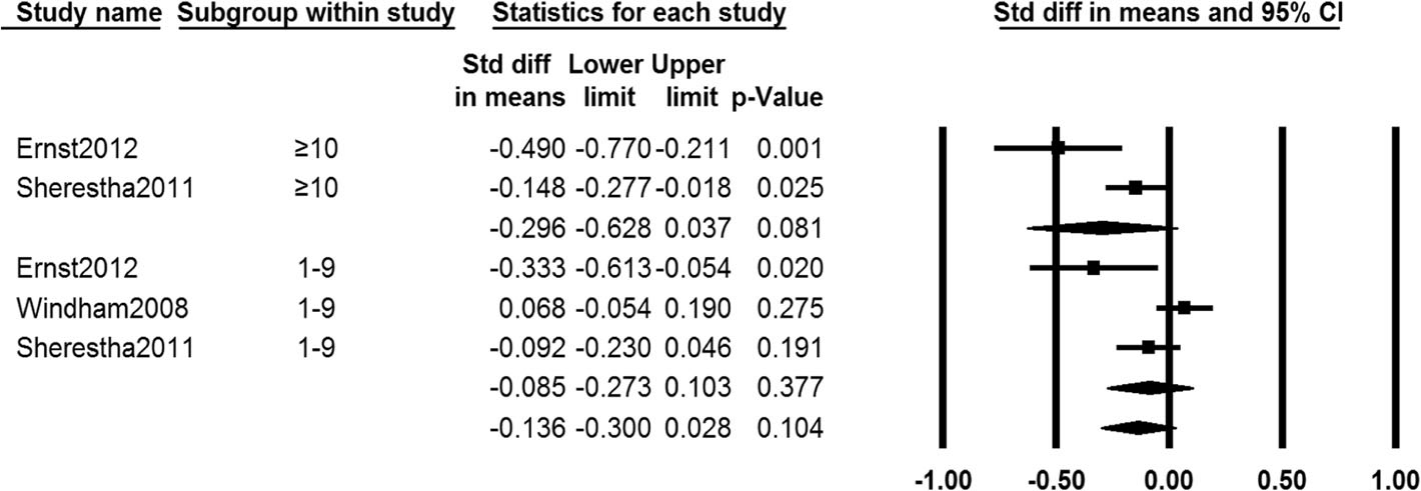
Forest plot for the age at menarche between different PTS exposure levels group and control group

Data on age at menarche of girls who exposed to different PTS levels were provided in three studies [23, 30, 31]. The subgroup analysis showed that there were no significant differences between different PTS exposure levels and control groups in age at menarche (SMD − 0.085, 95% CI − 0.273 to 0.103, 2633 girls, for 1–9 cigs/day subgroup; SMD − 0.296, 95% CI − 0.628 to 0.037, 1556 girls, for ≥ 10 cigs/day subgroup, *I*^2^ = 77.730%). The random effects model was adopted because of heterogeneity among studies (Fig. 3).

Behie 2015 [34] mentioned that with non-smoking mothers used as the reference level, mothers who reported smoking cigarettes ‘most days’ during gestation showed an HR of 1.40 (95% CI 1.10–1.79), which suggested that prenatal smoke exposure increase the chance of an earlier age at menarche.

#### Number of girls with early menarche

##### Number of girls with early menarche in PTS or ETS group

**Fig. 4:**
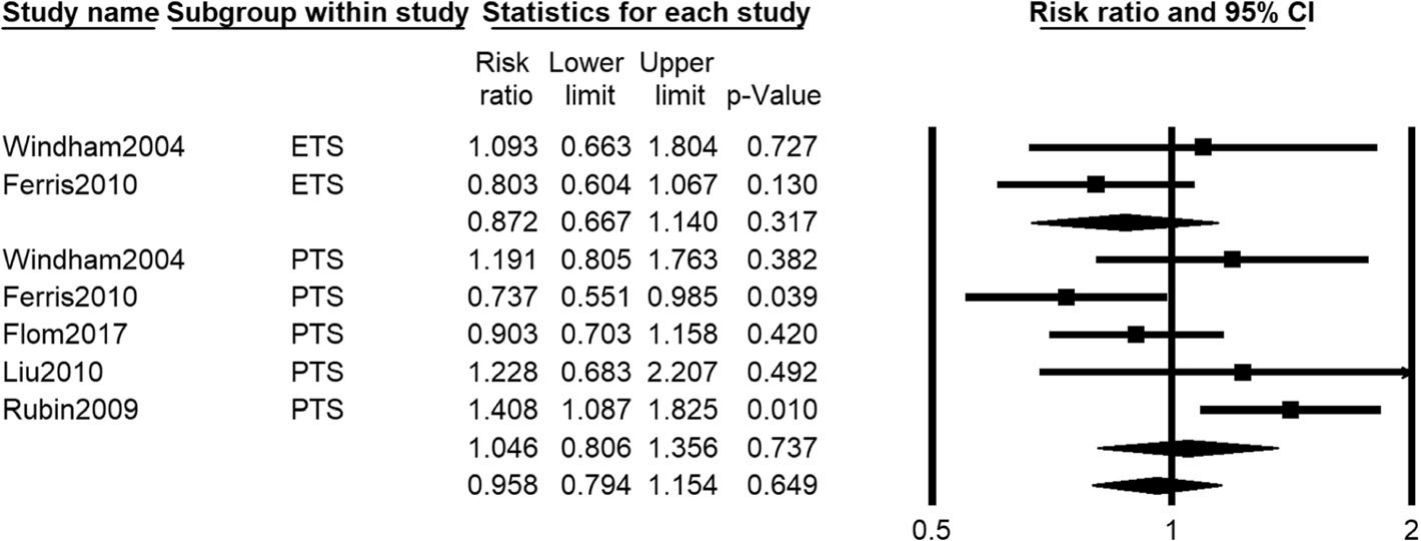
Forest plot for the number of girls with early menarche between the PTS or ETS group and control group

Data on number of girls with early menarche who exposed to PTS or ETS were provided in five studies [30, 32, 37, 41, 48]. The pooled data showed that number of girls with early menarche under PTS or ETS exposure (RR 0.649, 95% CI 0.794 to 1.154, *I*^2^ = 60.541%, 5819 girls) was not significantly different from that in control group, and similar results were found in PTS subgroup (RR 0.737, 95% CI 0.806 to 1.356, *I*^2^ = 68.362%, 5292 girls) and ETS subgroup (RR 0.872, 95% CI 0.667 to 1.140, *I*^2^ = 9.575%, 527 girls). Random effects model was adopted in the meta-analysis (Fig. 4).

For the five studies reporting PTS exposure, we conducted the subgroup analysis by the definition of early menarche. We found that number of girls in subgroup of early menarche defined as ≤ 11 years (RR 1.377, 95% CI 1.086 to 1.745, *I*^2^ = 0.000%, 3321 girls) was significantly more in PTS exposure group than control group, while no significantly difference was found in number of girls in subgroup of early menarche defined ≤ 12 (RR 0.900, 95% CI 0.708 to 1.145, *I*^2^ = 46.688%, 1971 girls) between two group (see Additional file 2: Figure S2).

And Behie 2015 [34] indicated the number of girls reached menarche (RR 1.276, 95% CI 1.154 to 1.411, *P* = 0.000, 1493 girls) was significantly higher in PTS exposure group than that in control group.

##### Number of girls with early menarche exposed to both PTS and ETS

**Fig. 5:**
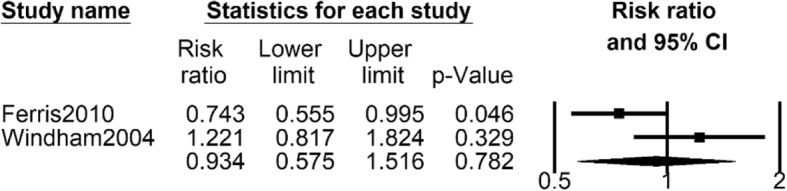
Forest plot for the number of girls with early menarche between both PTS and ETS group with control group

Two studies [30, 32] provided data about number of girls with early menarche exposed to both PTS and ETS. The pooled data showed that number of girls with early menarche with both PTS and ETS exposure (RR 0.934, 95% CI 0.575 to 1.516, *I*^2^ = 74.0%, 180 girls) was not significantly different from that in control group, and the random effects model was adopted because of the high heterogeneity (Fig. 5).

##### Number of girls with early menarche in different PTS exposure levels

**Fig. 6:**
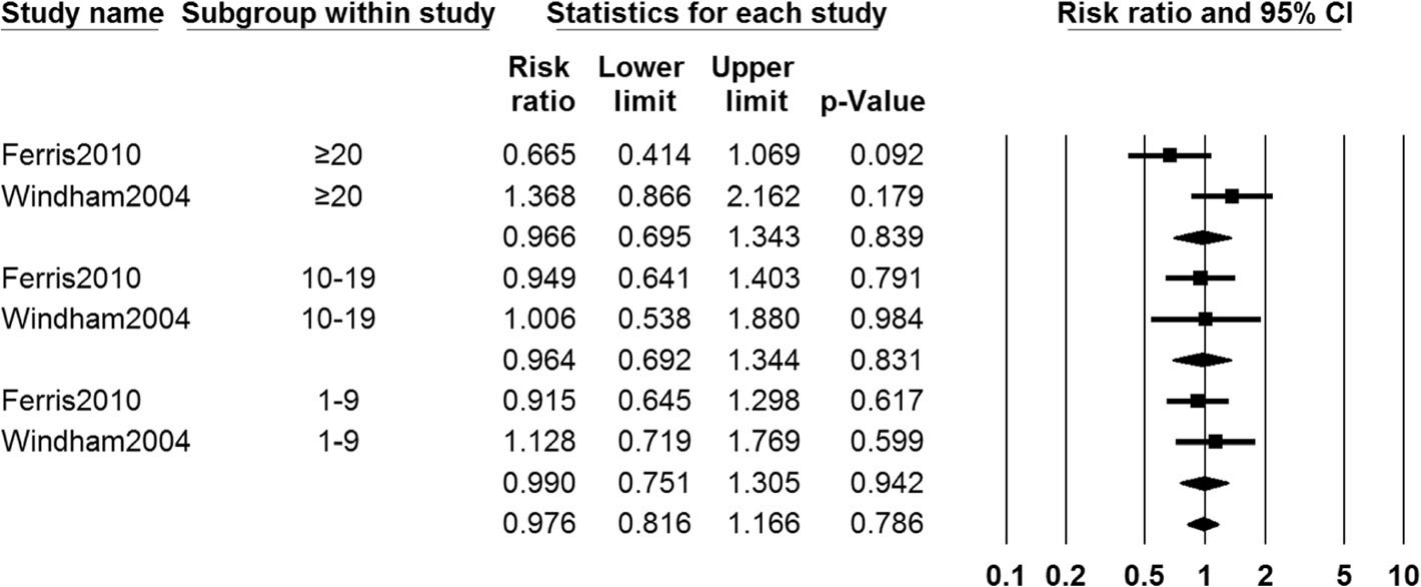
Forest plot for the number of girls with early menarche between different PTS exposure levels group and control group

Two studies [30, 32] provided data about number of girls with early menarche who exposed to different PTS levels. The pooled data showed that there were no significant differences between different PTS exposure level groups and control groups in the number of girls with early menarche (RR 0.990, 95% CI 0.751 to 1.305, 644 girls, for 1–9 cigs/day subgroup; RR 0.964, 95% CI 0.692 to 1.344, 479 girls, for 10–19cigs/day subgroup; RR 0.966, 95% CI 0.695 to 1.343, 575 girls, for ≥ 20 cigs/day subgroup, *I*^2^ = 3.17%). The fixed-effects model was adopted (Fig. 6).

#### Puberty index in boys

Three studies [29, 39, 46] reported different puberty index of boys exposed to PTS. In Håkonsen 2013 [29], age at acne, voice break, regular shaving, and first nocturnal emission in boys with PTS exposure were not significantly different from that in control group in either of the four exposure level groups (Table 3), and similar results were found in number of boys with early growth of penis and early pubic hair development in Ravnborg 2011 [46]. But the number of boys with early voice break with PTS exposure (RR 1.34, 95% CI 1.29 to 1.40, 2478 boys) was significantly higher than control group (Table 4).

**Table 3:** Mean difference (MD) and 95%CI of age at four puberty index of boys with different PTS exposure levels in Håkonsen 2013

Puberty index	PTS exposure level	MD	95% CI
Age at acne	Past smoker	0.10	0.08, 0.28
	1–9 cigs/day	0.10	− 0.05, 0.25
	10–14 cigs/day	− 0.10	− 0.28, 0.08
	≥ 15 cigs/day	− 0.20	− 0.43, 0.03
Age at voice break	Past smoker	0.00	− 0.17, 0.17
	1–9 cigs/day	0.10	− 0.04, 0.24
	10–14 cigs/day	− 0.10	− 0.27, 0.07
	≥ 15 cigs/day	− 0.10	− 0.32, 0.12
Age at regular shaving	Past smoker	0.10	− 0.08, 0.28
	1–9cigs/day	0.00	− 0.15, 0.15
	10–14 cigs/day	0.00	− 0.18, 0.18
	≥ 15 cigs/day	0.10	− 0.13, 0.33
Age at first nocturnal emission	Past smoker	0.00	− 0.22, 0.22
	1–9 cigs/day	0.10	− 0.09, 0.29
	10–14 cigs/day	− 0.10	− 0.32, 0.12
	≥ 15 cigs/day	− 0.20	− 0.49, 0.09

**Table 4:** Risk ratio (RR) and 95%CI of number of boys with early puberty index development with PTS exposure in Ravnborg 2011

Puberty index	RR	95% CI	*p*
Number of boys with early voice break	1.34	1.29, 1.40	0.00
Number of boys with early growth of penis	0.99	0.94, 1.04	0.64
Number of boys with early pubic hair development	0.99	0.93, 1.04	0.60

Fried 2001 [39] reported that an ANOVA of the age at which the boy’s voices began to change indicated that the higher level of prenatal smoking was associated with earlier onset of this pubertal indicant [*F*(2.61) = 7.82, *P* < 0.01], and the onset of shaving has the same trend emerged with as the voice change data.

## Discussions

For all we know, this is the first systematic review and meta-analysis of the relationship between PTS and/or ETS exposure and puberty timing of both girls and boys. We found two reviews about PTS and puberty; one of which conducted a qualitative description, reporting a hypothesis of increasing risk of puberty onset in boys and girls with PTS exposure. The other meta-analysis about PTS and age of menarche in girls suggested that PTS exposure may accelerate onset of age of menarche. Findings in these two reviews were relatively coincident with our results of PTS and age of menarche in girls or voice break onset in boys. Comparing with the previous two reviews, our study has a wider scope of tobacco exposure mode (both PTS and ETS) and included both genders. Besides, not only age of menarche, but also number of girls with early puberty events, number of boys with early puberty events, age at various puberty events were analyzed in this study. In addition, studies conducted in both developing and developed countries were included in the review, involving Asian population.

In present study, we found that PTS was possibly and negatively associated with age of menarche in girls, which suggested that mothers smoking during pregnancy may accelerate the onset of menarche of daughters. But this result was not stable enough, when removing out Maisonet 2010 [38], it turned to no association. Given the high heterogeneity, studies in PTS group were analyzed by cohort type in the subgroup analysis, and the heterogeneity dropped from 67.069 to 0.000% in prospective cohort subgroup, which suggested that study design may be one of the main sources of heterogeneity in PTS group. Prospective cohort study has a better demonstrated effectiveness which shows that the conclusion of early menarche with PTS is well founded.

While the results showed that there is no statistical significance on association between ETS and age of menarche, which may not necessarily mean that there is no relationship between them. As fewer studies reported ETS and puberty timing, the exact conclusion cannot be given exactly, which calls for more high-quality studies to confirm the relationship.

There was no strong evidence that PTS or ETS is associated with the number of girls with early menarche. The possible reason may be the differences in cut-offs for early menarche. There were four definitions for early menarche in the five studies [32, 35, 37, 41, 48]: ≤ 12, < 12, ≤ 11, < 11 years respectively. When early menarche defined as ≤ 11 years, the number of girls with early menarche in PTS group was significantly more than that in control group, and the heterogeneity also decreased when we conducted subgroup analysis by definition of early menarche.

Results from three studies on the association between PTS and puberty development in boys showed consistent results. Fried 2001 [39] reported an earlier age of voice break and shaving onset among boys exposed to PTS, which was based on a small cohort without confounder adjustment. The large retrospective study by Ravnborg 2011 [46] showed that exposed boys experienced an earlier voice break without confounder adjustment. The latest prospective study by Håkonsen 2012 [29] reported tendencies toward earlier age at acne, voice break, regular shaving, and first nocturnal emission, which indicated that boys exposed to PTS had earlier onset of puberty. However, no statistically significant differences were found with several important potential confounders adjusted. And no data provided can be used in meta-analysis in above studies. Therefore, the association between PTS exposure and puberty in boys could not be inferred. More studies reporting unified and complete outcome measures need to be conducted in boys to confirm the association.

The mechanism by which smoking exposure influence puberty timing is not clear enough yet. While 4000 chemicals contained over cigarettes, nicotine reduced blood flow to the placenta and fetus in pregnant smokers [18], and heavy metal cadmium led a retardation of trophoblastic outgrowth and development of placental [19]. Studies of animals and humans suggested that PTS exposure alter production of sex hormones and gonadotrophins [22–24], which are all crucial chemicals in puberty onset. For females, PTS exposure may potentially impact primordial follicle number at puberty and uterine volume [20]. And both PTS and ETS exposure to nicotine resulted in delayed ovarian dysfunction in adult female offspring [21]. For males, several large studies reported that moderate or heavy smoking in pregnancy reduced the testis size and sperm count of male offspring in adult by 20–40% [49, 50].

Studies included in the systematic review were conducted in four continents, namely, North America, Europe, Oceania, and Asia with multiethnic. To a certain extent, the results of this systematic review remind parents of the tobacco exposure effect on their children.

We carried out the research in strict accordance with the criterion of systematic review. However, there are still some limitations. First, though we tried to contact with the original authors to obtain necessary data of the included studies, six studies failed to obtain data required for meta-analysis. Among them, three [29, 39, 46] reported different puberty events of boys, respectively, and three [34, 45, 47] reported age of menarche did not provide sufficient data for meta-analysis. Failure to merge data above may lead to inaccuracy of results. Besides, one potential study in Czech was excluded, which may lead to selection bias.

Second, the assessment of methodology quality showed that scores of comparability and adequacy of follow-up of cohorts were relatively low; therefore, future research should pay more attention to these above aspects. Third, the main outcome measure of girls was menarche onset. Other outcomes cannot be conducted in meta-analysis due to few reports of tanner stage, inconsistent standard of division of staging and early onset. Meanwhile, reports of exposure levels were also limited and had different division standard, which made it difficult to find the dose-effect relationship.

Fourth, heterogeneity that comes from criteria of smoking exposure levels, race diversity, regional diversity, and sample size difference could not be analyzed in the current analysis. Fifth, few included studies reported the association of puberty development and PTS or ETS in boys, therefore need more studies to confirm this association. Finally, 4 out of 16 prospective cohort studies we included have not used the onset of outcome as the end of follow-up, so that children who have not occurred onset of puberty were excluded from the data analysis in these studies, which may lead to the loss of information and affect the accuracy of the results.

## Conclusion

In summary, the findings of this systematic review suggested that PTS exposure possibly decrease age of menarche of girls; there was still instability. No association were identified between ETS or different PTS levels with age of menarche, or PTS and/or ETS with number of girls with early puberty, or different PTS levels with number of girls with early puberty. As for boys, relationship between puberty timing with PTS or ETS remains to be further investigated with more high-quality cohort studies. Future studies should also provide appropriate and comprehensive outcome measures using unified definition to classify early or normal puberty for better comparison.

## Additional files

Additional file 1: **Figure S1.** Forest plot of dividing PTS group by cohort categories. (DOC 75 kb)

Additional file 2: **Figure S2.** Forest plot of dividing PTS group by the definition of early menarche. (DOC 69 kb)

## Abbreviations

CIs: Confidence intervals
ETS: Childhood environment tobacco smoke
PTS: Prenatal tobacco smoke
RR: Relative risk
SMD: Standardized mean difference
