# Supplementary figures and images for: Association of prenatal and childhood environment smoking exposure with puberty timing: a systematic review and meta-analysis
Source: Environ Health Prev Med. 2018 Jul 18;23:33. doi: 10.1186/s12199-018-0722-3 (PMC6052528; doi:10.1186/s12199-018-0722-3)

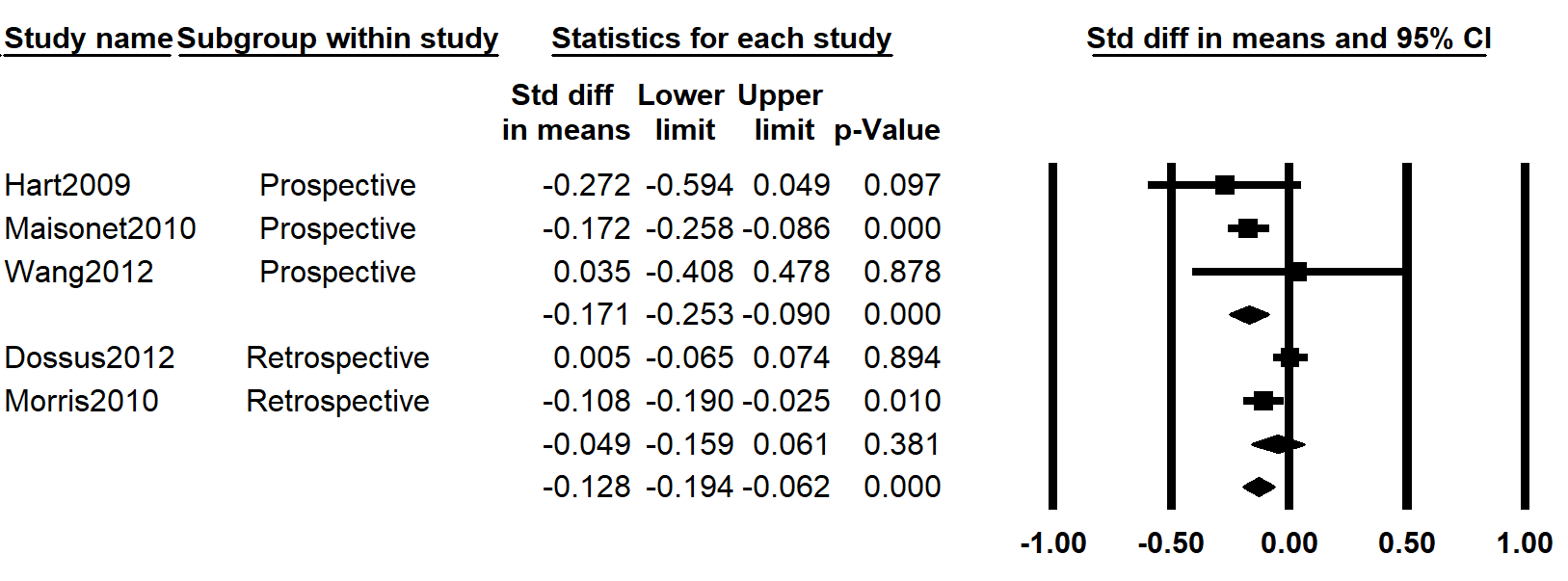


**Figure S1.**  Forest plot of dividing PTS group by cohort categories.

Supplement: Supplementary file 1 — Figure S1. Forest plot of dividing PTS group by cohort categories. (DOC 75 kb) [file 12199_2018_722_MOESM1_ESM.doc]

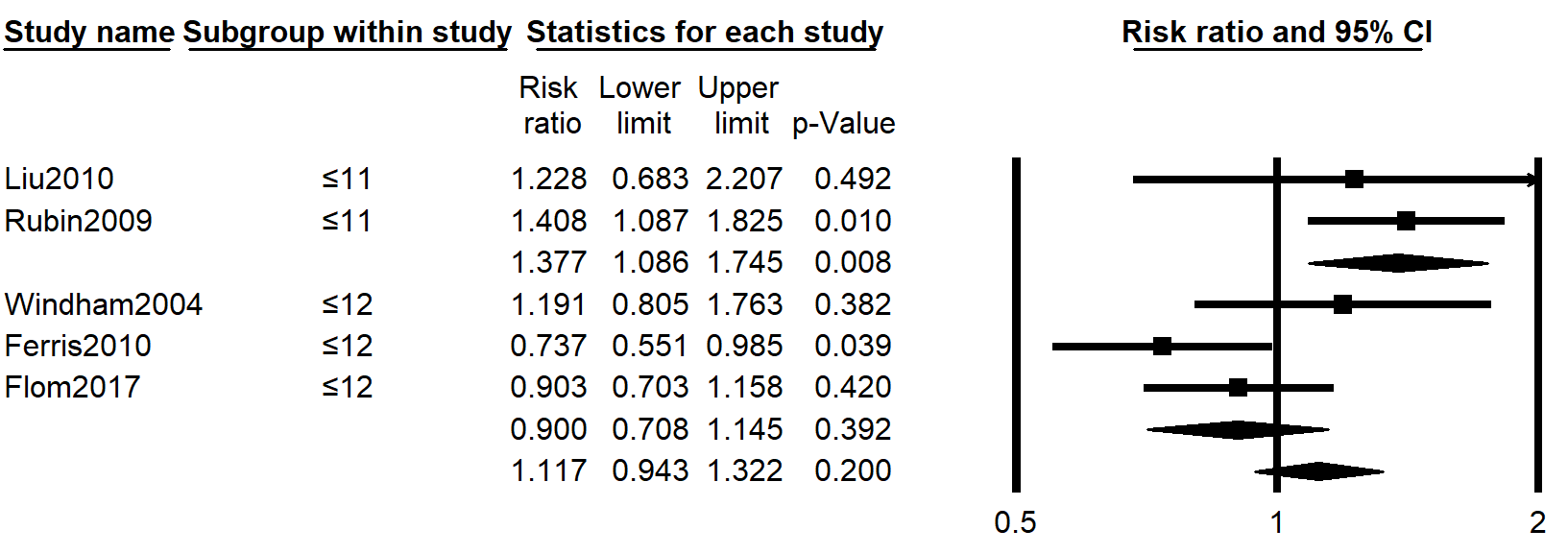


**Figure S2.** Forest plot of dividing PTS group by the definition of early menarche.

Supplement: Supplementary file 2 — Figure S2. Forest plot of dividing PTS group by the definition of early menarche. (DOC 69 kb) [file 12199_2018_722_MOESM2_ESM.doc]
